# DGK-θ: Structure, Enzymology, and Physiological Roles

**DOI:** 10.3389/fcell.2016.00101

**Published:** 2016-09-14

**Authors:** Becky Tu-Sekine, Hana L. Goldschmidt, Daniel M. Raben

**Affiliations:** Department of Biological Chemistry, The Johns Hopkins University School of MedicineBaltimore, MD, USA

**Keywords:** diacylglycerol kinase, phosphatidic acid, regulation, synaptic vesicle cycle, interfacial enzymology

## Abstract

Diacylglycerol kinases (DGKs) are a family of enzymes that catalyze the ATP-dependent phosphorylation of diacylglycerol (DAG) to phosphatidic acid (PtdOH). The recognition of the importance of these enzymes has been increasing ever since it was determined that they played a role in the phosphatidylinositol (PtdIns) cycle and a number of excellent reviews have already been written [(see van Blitterswijk and Houssa, 2000; Kanoh et al., 2002; Mérida et al., 2008; Tu-Sekine and Raben, 2009, 2011; Shulga et al., 2011; Tu-Sekine et al., 2013) among others]. We now know there are ten mammalian DGKs that are organized into five classes. DGK-θ is the lone member of the Type V class of DGKs and remains as one of the least studied. This review focuses on our current understanding of the structure, enzymology, regulation, and physiological roles of this DGK and suggests some future areas of research to understand this DGK isoform.

## Structure

**Figure 1:**
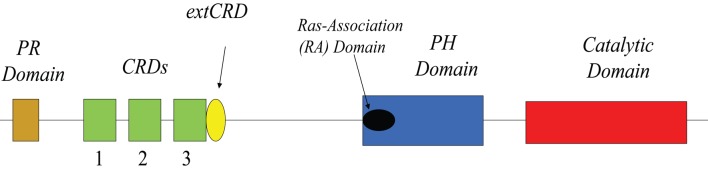
**Linear domain structure of DGK-θ**.

Arguably, our lack of knowledge about the three dimensional structure of mammalian DGKs remains as one of our most difficult challenges. Currently, our knowledge of the structural features of DGKs is essentially limited to the primary sequence of these enzymes (Figure 1). As noted in many excellent reviews (for example, see van Blitterswijk and Houssa, 2000; Kanoh et al., 2002; Mérida et al., 2008; Shulga et al., 2011) DGK-θ's major distinguishing feature in this regard is that it possess three, instead of two, C1 domains. Like other DGKs, the C1 domains of DGK-θ is homologous to PKC C1A and C1C domains with an extended region closest to the presumed catalytic domain near the C terminus. Despite the temptation to assume these domains bind DAG, their precise role remains unclear. Such caution is supported by the observation that the C1 domains of DGK-θ does not bind phorbol esters (Shindo et al., 2003). Further, the structural studies of Hurley and colleagues showed clear differences between these domains and those found in PKCs (Hurley et al., 1997). As suggested previously (Shulga et al., 2011), these data suggest that these DGK-θ domains may bind other lipids or participate in protein-protein interactions. Such a notion is supported by the evidence showing the C1 domain of another DGK, DGKζ, mediates the interactions with β-arrestins (Nelson et al., 2007) and Rac1 (Yakubchyk et al., 2005). We should note, that there is some evidence that this domain may be involved in membrane association in response to activation of some G-protein coupled receptors (van Baal et al., 2005).

Another feature of DGK-θ is the presence of a sequence with homology to Ras binding domains which is termed the Ras-Association (RA) domain (Houssa et al., 1997). It is important to note, however, that an *in silico* analyses based on binding energies predicts that the RA domain of DGK-θ probably does not bind Ras (Kiel et al., 2005). It is interesting, in this regard, that DGK-θ has been shown to bind a different small GTPase, active (GTP-bound) RhoA, and this association inhibits the kinase activity (Houssa et al., 1997). As with the C1 domains, however, it is not clear that RhoA binds specifically to this domain either and it is equally unclear as to whether RhoA is a regulator under various physiological conditions (see Regulation below).

Another major distinguishing feature of DGK-θ is the presence of a proline/glycine-rich region near its N-terminus. It is interesting to speculate that this region, may bind to proteins containing an SH3 domain given the presence of a pXPXXP motif (Yu et al., 1994). Further, a splice form of DGKδ, DGKδ2, also contains a proline-rich domain which is believed to play a role in regulating its membrane association in response to certain stimuli (Takeuchi et al., 2012). While these data are interesting, the precise role for the proline/glycine-rich domain in DGK-θ has not been fully examined.

The above clearly highlights the need for a greater understanding of the structural features of DGK-θ as well as other DGKs. Much of our structural understanding of these enzymes comes from studies of two prokaryotic DGKs. The first is a DGK found in the gram-negative organism Escherichia coli. This enzyme is different from the mammalian enzymes in that in contains three membrane spanning domains and functions as a trimer (see Van Horn and Sanders, 2012). Another DGK, found in gram-positive organisms such as *Staphylococcus aureus*, is a soluble enzyme with 15–18% homology to human DGKs (Miller et al., 2008). The structural studies of this enzyme showed the active site appears to form at a homodimer interface. Further, the data suggest a putative DAG substrate recognition loop that may provide an important difference between DGKs and related enzymes such as YegS and NAD kinases that are not bona fide DGKs (Bakali et al., 2007; Nichols et al., 2007; see Miller et al., 2008). Importantly, there is a clear need for clear structural studies on eukaryotic enzymes to determine not only the catalytic site, but also the structure of likely regulatory domains, such as the N-terminus, which are not present in the prokaryotic enzymes.

Progress in understanding the structure of eukaryotic DGKs including DGK-θ has been hampered by our inability to purify a sufficient amount of enzyme in a uniform, monodisperse solution that would be amenable to classic methodologies such as NMR and X-ray crystallography. So far, only the SAM (sterile alpha motif) domain of DGKδ1 has been determined by x-ray crystallography at a resolution of 2.9 angstrom (Harada et al., 2008). This study indicated that oligomerization of the SAM domain in a head to tail orientation leads to the cytosolic sequestration, and inhibition, of this enzyme. In addition to this structure, a solution structure of the C1 domain of DGKδ1, and although a structure of DGKα has been deposited in a structure database, it has not yet been published. We are clearly in need of new and creative approaches to determine the structure of DGK-θ and other DGK isoforms.

## Enzymology

DGK-θ belongs to a class of enzymes referred to as interfacial enzymes. These enzymes are generally soluble and have at least one insoluble substrate that is present in membranes or lipid aggregates. As a result, the enzymology of these enzymes, including DGK-θ, cannot be approached as is done for soluble enzymes with soluble substrates. Binding to membranes is often independent of catalysis and enzyme and substrate approach each other via diffusion within a membrane or lipid aggregate. Further complicating the analyses is the amount of time the enzyme resides on the interface, i.e., the enzyme's processivity, which may vary with the composition or architecture of the interface. We recently published a protocol (Tu-Sekine and Raben, submitted) that outlines an approach to the enzymology of these enzymes.

Analyses of DGK-θ present in cytosolic lysates illuminated two interesting aspects of its regulation (Tu-Sekine et al., 2006, 2007; Tu-Sekine and Raben, 2010). First, the enzyme is sensitive to both the bulk as well as surface concentration of the DAG substrate (Tu-Sekine et al., 2007). Second, the apparent Km (Km(app)) of the enzyme in these lysates was dependent on prior treatment of the cells. When DGK-θ was overexpressed in fibroblasts, the Km(app) of the enzyme for diacylglycerol (DAG) was increased in lysates obtained from mitogen-induced cells. The Km(app) then decreased with increasing bulk concentration of DAG. Additionally, the apparent Vmax (Vmax(app)) was increased in response to increases in the product, PtdOH (Tu-Sekine et al., 2007). It is noteworthy that the activation of DGK-θ in response to PtdOH occurs at 6-fold lower surface and bulk concentrations of DAG than observed when the activation occurs in response to phosphatidyserine (PtdSer). Importantly, the significant changes in the *K*_M_ and *V*_max_ values were observed only when physiological levels of both PtdOH and PtdSer were reached (Tu-Sekine et al., 2007).

To fully understand the enzymology, studies must be conducted on purified enzymes. One of the surprising discoveries when isolated DGK-θ was analyzed was that the enzyme is not fully active when purified. Full activity was observed when the purified enzyme was assayed in the presence of proteins rich in polybasic residues. In the presence of such polybasic cofactors, which directly interact with the enzyme, the apparent K(m) of the enzyme for DAG at the membrane surface (K(m)((surf))) was decreased These cofactors also decreased the Km for ATP. Furthermore, the cofactors synergistically increase the activity 10- to 30-fold in the presence of acidic phospholipids which were known to enhance enzyme activity (below).

There has been considerable interest in gaining a complete understanding of the enzymology and catalytic mechanism of DGK-θ and related kinases. In 2002, an interesting review appeared that highlighted the possibility that the similarity in phosphate-donor-binding sites may lead to a similar phosphorylation mechanism for four enzymes; DGKs, sphingosine kinases, NAD kinases, and phosphofructo-6-kinase (Labesse et al., 2002). Clearly, a confirmation of this hypothesisfor DGKs requires a comprehensive understanding of their structures. For example, it is possible that DGK-θ and other DGKs, act as a dimer similar to that suggested for DGKB in *Staphylococcus aureus* (Miller et al., 2008). Indeed, this may be a common paradigm for lipid kinases as another lipid kinase, sphingosine kinase 1, has also been suggested to function as a dimer (see Adams et al., 2016). As with enzymes in general, a complete understanding of DGK-θ's enzymology and catalytic mechanism will also provide critical information for the design of specific DGK-θ inhibitors.

## Regulation

As noted above, DGK-θ is very sensitive to the bulk and surface concentration of the DAG substrate. This provides a mechanism for regulating the enzyme in response to increases in DAG that accompany many signaling pathways. Additionally, as noted, DGK-θ activity is increased in response to PtdSer as well as PtdOH. Taken together, the data suggest that when DAG is generated in a signaling pathway, the Km(app) decreases and as the DAG concentration and PtdOH concentrations increase, the enzyme becomes more efficient. This could help explain the very transient nature of DAG increases in signaling pathways.

In addition to variations in membrane lipids, there are strong data supporting a role for protein-protein interactions that regulate DGK-θ activity. In 1999, Houssa et al. reported that activated (GTP-bound) RhoA associated with DGK-θ and inhibited its activity. Consistent with these data, Nurrish and colleagues have provided some fascinating data showing the homolog of DGK-θ in *C. elegans*, DGK-1, is regulated by RhoA and to modulate acetylcholine release at neuromuscular junctions (Nurrish et al., 1999; McMullan et al., 2006). As indicated above, there is a Ras-associating (RA) domain within the PH domain of DGK-θ and it has been tempting to speculate that this region was responsible for RhoA binding. Los et al., however, showed that RhoA bound to the C-terminal portion of the catalytic domain although they did not completely rule out another interaction with the RA domain (Los et al., 2004). While the precise RhoA binding regions have not been fully identified, a potential important role for these interactions is given support from the observations that other DGKs may show similar associations. For example, Tolias et al. presented evidence that a DGK associated with Rac1 (Tolias et al., 1998). This enzyme, however, is unlikely to be DGK-θ as another study showed DGK-θ does not associate with Rac1 or cdc42 (Houssa et al., 1999).

The role of phosphorylation in regulating DGK-θ is unclear. Using lysates of Cos-7 cells, PKC-ε has been shown to phosphorylate DGK-θ *in vitro* and studies using A431 cells suggested that this phosphorylation may promote membrane association (van Baal et al., 2005). Akt has also been shown to associate with and stimulate DGK-θ catalytic activity. Consistent with this, PI-3 kinase regulates the translocation and activation of DGK-θ in rat mesenteric arteries although, importantly, the mechanism of this regulation is unclear and may not involve DGK-θ phosphorylation (Walker et al., 2001; Clarke et al., 2007). Additional studies are clearly required to define the precise role of DGK-θ phosphorylation in membrane association and catalytic activation.

**Figure 2:**
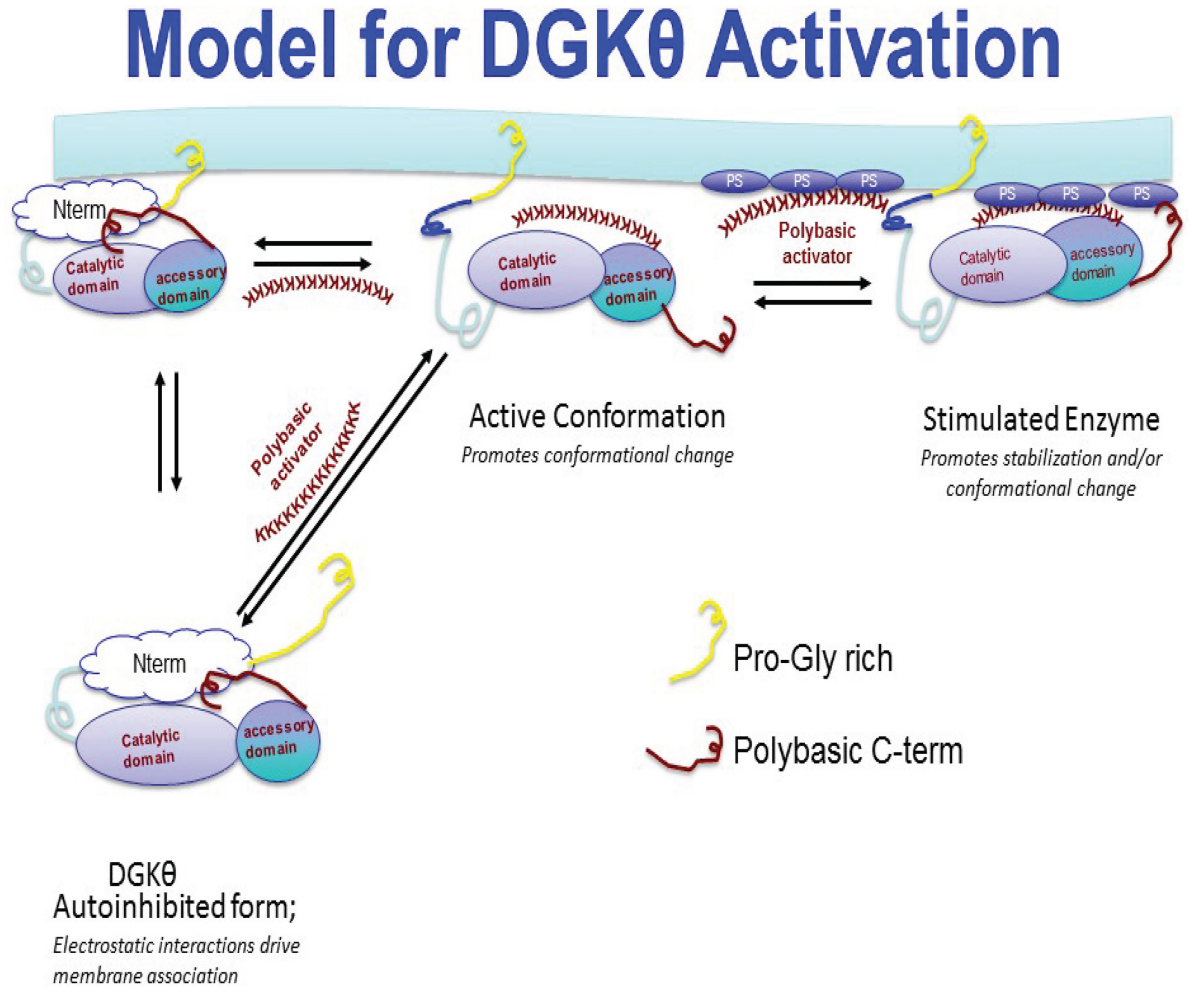
**Proposed model for DGK-θ membrane association and activation**. In this model, DGK-θ exists in an auto-inhibited state which can bind to membranes in the presence or absence of a protein activator containing a polybasic region. Following membrane binding, full activation is achieved when a triad complex forms composed of enzyme, polybasic protein, and phospholipid activator.

As indicated above, full activation of DGK-θ requires an acidic phospholipid and a polybasic cofactor. Additional analyses showed that while the acidic phospholipids recruit polybasic cofactors to the surface of artificial membranes, they do not affect the membrane association of DGK-θ suggesting interfacial association and catalytic activity are independently regulated. The data provide support for a model (Figure 2) in which DGK-θ exists as an auto-inhibited enzyme and that full interfacial activity requires a triad of enzyme, acidic lipid, and basic protein (Tu-Sekine et al., 2007). The enzyme is also likely to be modulated by the presence of RhoA or another small GTPase under physiological conditions. Importantly, the polybasic protein that represents the physiological regulator of DGK-θ in various tissues has/have not been identified and represents an important challenge in the field.

## Physiological roles

### Regulation of gene expression

DGK-θ shows a broad tissue distribution with a regulated expression profile (Ohanian and Ohanian, 2001; Topham, 2006; Ueda et al., 2013). These studies has shown it is present in various tissues that include brain, intestine, liver, kidney, and small arteries and our understanding of its physiological roles are beginning to emerge. One of the physiological roles identified for DGK-θ was as a modulator of gene expression (Li et al., 2007). Consistent with this, DGK-θ has been found to localize in the nucleus in some cells (Bregoli et al., 2001, 2002; Tabellini et al., 2003, 2004). In two immortalized neuronal cell lines (PC-12 and N2a) the enzyme has been found in speckle domains of the nucleus within a subpopulation of cells (Tabellini et al., 2003). As speckle domains are known to contain splicing factors, these data suggest that DGK-θ may play a role in RNA processing. In support of this notion, NGF (nerve growth factor) induces an increase in the level and activity of DGK-θ in the nucleus (Tabellini et al., 2003). Some of the most compelling evidence supporting a role for DGK-θ in modulating gene expression has been provided by Sewer and colleagues (Li et al., 2007; Cai and Sewer, 2013; Cai et al., 2014). They show DGK-θ plays a role in the activation of the nuclear receptor steroidogenic factor 1 (SF-1). Knock down of DGK-θ, but not DGK-ζ, or expression of a kinase-dead mutant of DGK-θ abrogated agonist-induced expression of SF-1-mediated genes (Li et al., 2007). This presents another exciting area for future investigations to illuminate the role of DGK-θ-mediated nuclear PtdOH production.

### Regulation of synaptic cycling and neurotransmission

Nine of the ten known mammalian DGKs are expressed in neurons (see Tu-Sekine and Raben, 2011; Ishisaka and Hara, 2014). This isoform has been shown to be expressed throughout the brain with the strongest expression found in the hippocampus and cerebellar cortex (Houssa et al., 1997; Tu-Sekine and Raben, 2011; Ueda et al., 2013; Ishisaka and Hara, 2014). Recently, interest in the roles and regulation of DGKs in the mammalian central nervous system (CNS) has grown (Tu-Sekine and Raben, 2011; Ishisaka and Hara, 2014). However, as all ten DGK isoforms are abundant in this tissue, determination of unique cellular functions for each DGK isoform has been difficult. DGK-θ is expressed early during brain during development (Ueda et al., 2013) and subsequently maintained through adulthood where it has been detected in the adult nervous system (Houssa et al., 1997; Tu-Sekine and Raben, 2011; Ueda et al., 2013; Goldschmidt et al., 2016). Initial studies implicating a role for DGK-θ in synapse function came from elegant work on the *C. elegans* homolog of DGK-θ, DGK-1 (Nurrish et al., 1999; McMullan et al., 2006). Nurrish et al. showed that loss-of-function mutations in DGK-1 potentiated acetylcholine at neuromuscular junctions. This increase appears to be due to hyper-stimulation of the DAG-binding vesicle-priming protein unc-13 (Nurrish et al., 1999; Miller et al., 1999; and see Kanoh et al., 2002; Mérida et al., 2008). Based on these data, we speculated DGK-θ might regulate a similar function at mammalian central synapses (Goldschmidt et al., 2016). We found DGK-θ localized to excitatory synapses where its kinase activity promotes retrieval of synaptic vesicles following neuronal activity. Acute depletion of DGK-θ using shRNAs or significantly impaired the recycling kinetics of SVs. The same effect was observed in neurons derived from mice in which DGK-θ was genetically ablated although the mice were overtly normal. Importantly, the SV defect could only be rescued expression of enzymatically active DGK-θ, but not a kinase-dead enzyme. The data presented not only establish a role for DGK-θ kinase activity in SV cycling, but further analyses showed DGK-θ is likely to be important for synaptic transmission when sustained neuronal activity is required (Goldschmidt et al., 2016).

### Pathophysiological roles

At the present time, there is very little data regarding the pathophysiological role of DGK-θ. As noted earlier, this enzyme has been shown to be regulated in mesenteric arteries. The data suggests that this enzyme may play a role in regulating blood pressure in response to noradrenalin but not angiotensin II (Walker et al., 2001; Clarke et al., 2007). Clearly, this could have important implications for treating hypertension or pathophysiological conditions in which hypertension is a complicating factor. Other data suggest that RhoA-mediated inhibition of DGK-θ mediates ischemia/reperfusion injury in liver by allowing for the elevation of DAG (Baldanzi et al., 2010). Given the potential role for DGK-θ in modulating SF1 (above), it is tempting to speculate that this enzyme may play a role in diseases associated with this transcription factor (El-Khairi and Achermann, 2012). Our recent studies suggest that DGK-θ may play a critical role during sustained or intense neuronal stimulation (Goldschmidt et al., 2016) and therefore, may play a key role in modulating seizures. Taken together, while there are intriguing data regarding the physiologivcal and pathophysiological roles of DGK-θ additional studies are clearly needed.

## Future directions

While we have learned quite a bit about DGK-θ, there is clearly much more to be discovered. Understanding the structure of DGK-θ will provide essential insights into its catalytic mechanism and regulation. Developing new strategies to elucidate the structure of this enzyme, and identification of physiologically relevant polybasic regulators will be key in our understanding of this enzyme. Such data will be critical in our efforts to develop specific inhibitors for this isozyme which are at present not available. The importance of such inhibitors will be highlighted as we learn more about the various physiological roles of DGK-θ. For example, if DGK-θ is indeed critical during intense neuronal stimulation, a specific inhibitor may be useful in the treatment pathophysiological conditions in which such stimulation plays an important role (e.g., epilepsy). Conversely, the enzyme may play a role under physiological conditions where rapid SV cycling is essential such as ribbon synapses of some sensory neurons. The future of research on DGK-θ promises to be exciting and impactful.

## Author contributions

All authors listed, have made substantial, direct and intellectual contribution to the work, and approved it for publication.

## Funding

DR is funded by the National Institutes of Health RO1NS077923.

### Conflict of interest statement

The authors declare that the research was conducted in the absence of any commercial or financial relationships that could be construed as a potential conflict of interest.

## References

[B1] Adams D. R. Pyne S. Pyne N. J. (2016). Sphingosine kinases: emerging structure-function insights. Trends Biochem. Sci. 41, 395–409. 10.1016/j.tibs.2016.02.007 27021309

[B2] Bakali H. M. Herman M. D. Johnson K. A. Kelly A. A. Wieslander A. Hallberg B. M.. (2007). Crystal structure of YegS, a homologue to the mammalian diacylglycerol kinases, reveals a novel regulatory metal binding site. J. Biol. Chem. 282, 19644–19652. 10.1074/jbc.M604852200 17351295

[B3] Baldanzi G. Alchera E. Imarisio C. Gaggianesi M. Dal Ponte C. Nitti M.. (2010). Negative regulation of diacylglycerol kinase theta mediates adenosine-dependent hepatocyte preconditioning. Cell Death Differ. 17, 1059–1068. 10.1038/cdd.2009.210 20057501

[B4] Bregoli L. Baldassare J. J. Raben D. M. (2001). Nuclear diacylglycerol kinase-θ is activated in response to α-Thrombin. J. Biol. Chem. 276, 23288–23295. 10.1074/jbc.M101501200 11309392

[B5] Bregoli L. Tu-Sekine B. Raben D. M. (2002). DGK and nuclear signaling: nuclear diacylglycerol kinases in IIC9 cells. Adv. Enzyme Regul. 42, 213–226. 10.1016/S0065-2571(01)00032-2 12123717

[B6] Cai K. Lucki N. C. Sewer M. B. (2014). Silencing diacylglycerol kinase-theta expression reduces steroid hormone biosynthesis and cholesterol metabolism in human adrenocortical cells. Biochim. Biophys. Acta 1841, 552–562. 10.1016/j.bbalip.2013.12.005 24369117 PMC4005595

[B7] Cai K. Sewer M. B. (2013). cAMP-stimulated transcription of DGKtheta requires steroidogenic factor 1 and sterol regulatory element binding protein 1. J. Lipid Res. 54, 2121–2132. 10.1194/jlr.M035634 23610160 PMC3708362

[B8] Clarke C. J. Ohanian V. Ohanian J. (2007). Norepinephrine and endothelin activate diacylglycerol kinases in caveolae/rafts of rat mesenteric arteries: agonist-specific role of PI3-kinase. Am. J. Physiol. Heart Circ. Physiol. 292, H2248–H2256. 10.1152/ajpheart.01170.2006 17208990

[B9] El-Khairi R. Achermann J. C. (2012). Steroidogenic factor-1 and human disease. Semin. Reprod. Med. 30, 374–381. 10.1055/s-0032-1324720 23044873 PMC5159746

[B10] Goldschmidt H. L. Tu-Sekine B. Volk L. Anggono V. Huganir R. L. Raben D. M. (2016). DGKtheta catalytic activity is required for efficient recycling of presynaptic vesicles at excitatory synapses. Cell Rep. 14, 200–207. 10.1016/j.celrep.2015.12.022 26748701 PMC4715728

[B11] Harada B. T. Knight M. J. Imai S. Qiao F. Ramachander R. Sawaya M. R.. (2008). Regulation of enzyme localization by polymerization: polymer formation by the SAM domain of diacylglycerol kinase delta1. Structure 16, 380–387. 10.1016/j.str.2007.12.017 18334213

[B12] Houssa B. de Widt J. Kranenburg O. Moolenaar W. H. van Blitterswijk W. J. (1999). Diacylglycerol kinase theta binds to and is negatively regulated by active RhoA. J. Biol. Chem. 274, 6820–6822. 10066731 10.1074/jbc.274.11.6820

[B13] Houssa B. Schaap D. van der Wal J. Goto K. Kondo H. Yamakawa A.. (1997). Cloning of a novel human diacylglycerol kinase (DGKtheta) containing three cysteine-rich domains, a proline-rich region, and a pleckstrin homology domain with an overlapping Ras-associating domain. J. Biol. Chem. 272, 10422–10428. 9099683 10.1074/jbc.272.16.10422

[B14] Hurley J. H. Newton A. C. Parker P. J. Blumberg P. M. Nishizuka Y. (1997). Taxonomy and function of C1 protein kinase C homology domains. Protein Sci. 6, 477–480. 10.1002/pro.5560060228 9041654 PMC2143645

[B15] Ishisaka M. Hara H. (2014). The roles of diacylglycerol kinases in the central nervous system: review of genetic studies in mice. J. Pharmacol. Sci. 124, 336–343. 10.1254/jphs.13R07CR 24599142

[B16] Kanoh H. Yamada K. Sakane F. (2002). Diacylglycerol kinases: emerging downstream regulators in cell signaling systems. J. Biochem. (Tokyo). 131, 629–633. 10.1093/oxfordjournals.jbchem.a003144 11983067

[B17] Kiel C. Wohlgemuth S. Rousseau F. Schymkowitz J. Ferkinghoff-Borg J. Wittinghofer F.. (2005). Recognizing and defining true Ras binding domains II: *in silico* prediction based on homology modelling and energy calculations. J. Mol. Biol. 348, 759–775. 10.1016/j.jmb.2005.02.046 15826669

[B18] Labesse G. Douguet D. Assairi L. Gilles A. M. (2002). Diacylglyceride kinases, sphingosine kinases and NAD kinases: distant relatives of 6-phosphofructokinases. Trends Biochem. Sci. 27, 273–275. 10.1016/S0968-0004(02)02093-5 12069781

[B19] Li D. Urs A. N. Allegood J. Leon A. Merrill A. H. Jr. Sewer M. B. (2007). Cyclic AMP-stimulated interaction between steroidogenic factor 1 and diacylglycerol kinase theta facilitates induction of CYP17. Mol. Cell Biol. 27, 6669–6685. 10.1128/MCB.00355-07 17664281 PMC2099220

[B20] Los A. P. van Baal J. de Widt J. Divecha N. van Blitterswijk W. J. (2004). Structure-activity relationship of diacylglycerol kinase theta. Biochim. Biophys. Acta 1636, 169–174. 10.1016/j.bbalip.2003.11.008 15164764

[B21] McMullan R. Hiley E. Morrison P. Nurrish S. J. (2006). Rho is a presynaptic activator of neurotransmitter release at pre-existing synapses in *C. elegans*. Genes Dev. 20, 65–76. 10.1101/gad.359706 16391233 PMC1356101

[B22] Mérida I. Avila-Flores A. Merino E. (2008). Diacylglycerol kinases: at the hub of cell signalling. Biochem. J. 409, 1–18. 10.1042/BJ20071040 18062770

[B23] Miller D. J. Jerga A. Rock C. O. White S. W. (2008). Analysis of the *Staphylococcus aureus* DgkB structure reveals a common catalytic mechanism for the soluble diacylglycerol kinases. Structure 16, 1036–1046. 10.1016/j.str.2008.03.019 18611377 PMC2847398

[B24] Miller K. G. Emerson M. D. Rand J. B. (1999). Goalpha and diacylglycerol kinase negatively regulate the Gqalpha pathway in *C. elegans* [see comments]. Neuron 24, 323–333. 10.1016/S0896-6273(00)80847-8 10571227 PMC4703424

[B25] Nelson C. D. Perry S. J. Regier D. S. Prescott S. M. Topham M. K. Lefkowitz R. J. (2007). Targeting of diacylglycerol degradation to M1 muscarinic receptors by beta-arrestins. Science 315, 663–666. 10.1126/science.1134562 17272726

[B26] Nichols C. E. Lamb H. K. Lockyer M. Charles I. G. Pyne S. Hawkins A. R.. (2007). Characterization of *Salmonella typhimurium* YegS, a putative lipid kinase homologous to eukaryotic sphingosine and diacylglycerol kinases. Proteins 68, 13–25. 10.1002/prot.21386 17393457

[B27] Nurrish S. Ségalat L. Kaplan J. M. (1999). Serotonin inhibition of synaptic transmission: galpha(0) decreases the abundance of UNC-13 at release sites. Neuron 24, 231–242. 10.1016/S0896-6273(00)80835-1 10677040

[B28] Ohanian J. Ohanian V. (2001). Lipid second messenger regulation: the role of diacylglycerol kinases and their relevance to hypertension. J. Hum. Hypertens. 15, 93–98. 10.1038/sj.jhh.1001139 11317187

[B29] Shindo M. Irie K. Masuda A. Ohigashi H. Shirai Y. Miyasaka K.. (2003). Synthesis and phorbol ester binding of the cysteine-rich domains of diacylglycerol kinase (DGK) isozymes. DGKgamma and DGKbeta are new targets of tumor-promoting phorbol esters. J. Biol. Chem. 278, 18448–18454. 10.1074/jbc.M300400200 12621060

[B30] Shulga Y. V. Topham M. K. Epand R. (2011). M. Regulation and functions of diacylglycerol kinases. Chem. Rev. 111, 6186–6208. 10.1021/cr1004106 21800853

[B31] Tabellini G. Billi A. M. Falà F. Cappellini A. Evagelisti C. Manzoli L.. (2004). Nuclear diacylglycerol kinase-theta is activated in response to nerve growth factor stimulation of PC12 cells. Cell Signal. 16, 1263–1271. 10.1016/j.cellsig.2004.03.018 15337525

[B32] Tabellini G. Bortul R. Santi S. Riccio M. Baldini G. Cappellini A.. (2003). Diacylglycerol kinase-theta is localized in the speckle domains of the nucleus. Exp. Cell Res. 287, 143–154. 10.1016/S0014-4827(03)00115-0 12799190

[B33] Takeuchi M. Sakiyama S. Usuki T. Sakai H. Sakane F. (2012). Diacylglycerol kinase delta1 transiently translocates to the plasma membrane in response to high glucose. Biochim. Biophys. Acta 1823, 2210–2216. 10.1016/j.bbamcr.2012.08.019 22974639

[B34] Tolias K. F. Couvillon A. D. Cantley L. C. Carpenter C. L. (1998). Characterization of a Rac1- and RhoGDI-associated lipid kinase signaling complex. Mol. Cell Biol. 18, 762–770. 10.1128/mcb.18.2.762 9447972 PMC108787

[B35] Topham M. K. (2006). Signaling roles of diacylglycerol kinases. J. Cell Biochem. 97, 474–484. 10.1002/jcb.20704 16288460

[B36] Tu-Sekine B. Goldschmidt H. Petro E. Raben D. M. (2013). Diacylglycerol kinase theta: regulation and stability. Adv. Biol. Regul. 53, 118–126. 10.1016/j.jbior.2012.09.007 23266086 PMC3992713

[B37] Tu-Sekine B. Ostroski M. Raben D. M. (2006). Analysis of two diacylglycerol kinase activities in mixed micelles. Adv. Enzyme Regul. 46, 12–24. 10.1016/j.advenzreg.2006.01.018 16854454

[B38] Tu-Sekine B. Ostroski M. Raben D. M. (2007). Modulation of diacylglycerol kinase theta activity by alpha-thrombin and phospholipids. Biochemistry 46, 924–932. 10.1021/bi061170c 17223715

[B39] Tu-Sekine B. Raben D. M. (2009). Regulation of DGK-theta. J. Cell Physiol. 220, 548–552. 10.1002/jcp.21813 19472209

[B40] Tu-Sekine B. Raben D. M. (2010). Characterization of cellular DGK-theta. Adv. Enzyme Regul. 50, 81–94. 10.1016/j.advenzreg.2009.10.031 19914279 PMC3608514

[B41] Tu-Sekine B. Raben D. M. (2011). Regulation and roles of neuronal diacylglycerol kinases: a lipid perspective. Crit. Rev. Biochem. Mol. Biol. 46, 353–364. 10.3109/10409238.2011.577761 21539478

[B42] Ueda S. Tu-Sekine B. Yamanoue M. Raben D. M. Shirai Y. (2013). The expression of diacylglycerol kinase theta during the organogenesis of mouse embryos. BMC Dev. Biol. 13:35. 10.1186/1471-213X-13-35 24079595 PMC3850696

[B43] van Baal J. de Widt J. Divecha N. van Blitterswijk W. J. (2005). Translocation of diacylglycerol kinase theta from cytosol to plasma membrane in response to activation of G protein-coupled receptors and protein kinase C. J. Biol. Chem. 280, 9870–9878. 10.1074/jbc.M409301200 15632189

[B44] van Blitterswijk W. J. Houssa B. (2000). Properties and functions of diacylglycerol kinases. Cell Signal. 12, 595–605. 10.1016/S0898-6568(00)00113-3 11080611

[B45] Van Horn W. D. Sanders C. R. (2012). Prokaryotic diacylglycerol kinase and undecaprenol kinase. Annu. Rev. Biophys. 41, 81–101. 10.1146/annurev-biophys-050511-102330 22224599 PMC3575517

[B46] Walker A. J. Draeger A. Houssa B. van Blitterswijk W. J. Ohanian V. Ohanian J. (2001). Diacylglycerol kinase θ is translocated and phosphoinositide 3-kinase-dependently activated by noradrenaline but not angiotensin II in intact small arteries. Biochem. J. 353(Pt 1), 129–137. 10.1042/bj3530129 11115406 PMC1221550

[B47] Yakubchyk Y. Abramovici H. Maillet J. C. Daher E. Obagi C. Parks R. J.. (2005). Regulation of neurite outgrowth in N1E-115 cells through PDZ-mediated recruitment of diacylglycerol kinase zeta. Mol. Cell Biol. 25, 7289–7302. 10.1128/MCB.25.16.7289-7302.2005 16055737 PMC1190239

[B48] Yu H. Chen J. K. Feng S. Dalgarno D. C. Brauer A. W. Schreiber S. L. (1994). Structural basis for the binding of proline-rich peptides to SH3 domains. Cell 76, 933–945. 10.1016/0092-8674(94)90367-0 7510218

